# Anomaly detection in cropland monitoring using multiple view vision transformer

**DOI:** 10.1038/s41598-025-98405-1

**Published:** 2025-04-23

**Authors:** Xuesong Liu, Yansong Liu, He Sui, Chuan Qin, Yuanxi Che, Zhaobo Guo

**Affiliations:** 1 James Watt School of Engineering, University of Glasgow, Glasgow, G12 8QQ UK; 2 School of Intelligence Engineering, Shandong Management University, Jinan, 250357 China; 3 College of Aeronautical Engineering, Civil Aviation University of China, Tianjin, 300300 China; 4 Department of Infrastructure Engineering, University of Melbourne, Melbourne, VIC 3010 Australia; 5 Department of Computer Science, Xidian University, Xi’an, 710126 China

**Keywords:** Vision transformer, Anomaly detection, Low altitude, Cropland, Machine vision, Attention mechanism, Plant sciences, Engineering, Mathematics and computing

## Abstract

In recent times, the importance of low-altitude security, especially in agricultural surveillance, has seen a remarkable upswing. This paper puts forward a novel Internet of Drones framework tailored for low-altitude operations. Anomaly detection, which is pivotal for ensuring the integrity of the entire system, poses a substantial challenge. Such anomalies can range from unpredictable weather patterns in farmlands to unauthorized intrusions. To surmount this, a comprehensive deep learning pipeline is proposed in this study. It deploys a vision transformer model featuring a unique attention mechanism. The pipeline includes the meticulous collection of a vast array of normal and abnormal farmland images, followed by preprocessing to standardize data. Anomaly detection is then carried out, and the model’s performance is evaluated using metrics like sensitivity (92.8%), specificity (93.1%), accuracy (93.5%), and F1 score (94.1%). Comparative analysis with state-of-the-art algorithms reveals the superiority of the proposed model. In the future, this study plans to explore integrating data from thermal, infrared, or LIDAR sensors, enhance the interpretability of the vision transformer model, and optimize the deep learning pipeline to reduce computational complexity.

## Introduction

In the realm of low-altitude security, especially in cropland monitoring, anomaly detection has emerged as a crucial aspect^1–4^. Anomaly detection in this context refers to the identification of any deviation from normal patterns in the cropland environment. These anomalies can range from irregular crop growth due to pests, diseases, or adverse weather conditions to unauthorized intrusions. Detecting such anomalies at an early stage is vital for ensuring crop health, maximizing yields, and maintaining security in agricultural areas.

In the past, various approaches have the potentials to address the anomaly detection in cropland monitoring. On one hand, the machine-learning algorithms have shown promising performance in anomaly detection. Yang et al.^5^ conducted a study on the use of a wireless sensor system for orchard management. Fent et al.^6^ designed an automated apple orchard monitoring system using the Internet of Things (IoT) to minimize resource use, enhance apple quality, and provide comprehensive data. The research^7^ presented a robotic platform specifically developed to monitor the state of plants. To address the monitoring difficulties encountered by apple orchards, Ref.^8^ devised a wireless sensor-driven system for monitoring apple orchards. The study^9^ introduced an algorithm to optimize drone functionality, addressing the fragility issue caused by high-weight functionalities in existing systems. It enhances drone performance across various paths by analyzing radial functions, data transmission coverage, and incorporating motion signatures and a special identification system. Then, the work of Ref.^10^ presented a method using cascading k-means clustering and the decision tree method C4.5, for classifying anomalous and typical computer network operations. Recently, Shitharth Selvarajan^11^ investigated the development in bio-inspired optimization techniques, analyzing their unique characteristics, optimization performance, and operational paradigms. It demonstrates their revolutionary potential in solving complex engineering problems. On the other hand, the deep learning methods have shown favorable results in the field of anomaly detection. Convolutional networks (ConvNets) that include multi-scale and hierarchical architectures have significantly influenced the development of object detection^12^. In their study, Grignaffini et al.^13^ introduced a convolutional neural network (CNN) model that incorporated handcrafted texture features of dermoscopic images as supplementary input during the training phase. He et al.^14^ suggested a method for constructing a high-performance framework for monitoring aberrant ECG rhythms, which effectively reduces the amount of data transmission. It offers a more effective hardware implementation and decreases the usage of hardware resources in comparison to current models. Zeng et al.^15^ introduced a Hierarchical Spatio-Temporal Graph Convolutional Neural Network as a solution for detecting anomalies in movies.

Despite the progress in related research, several research gaps remain. Existing machine-learning algorithms for anomaly detection in low-altitude airspace, especially those related to cropland monitoring using the Internet of Drones (IoD), struggle to efficiently handle the vast amounts of data generated. Many current methods rely on ConvNets, which may miss long-range relationships in the data. Additionally, there is a lack of comprehensive frameworks that can effectively utilize the multiple view (multi-view) data obtained from drones in cropland monitoring. Recently, since Vaswani et al. first proposed the transformer model for natural language processing (NLP) in their work^16^, deep learning models based on transformers have been extensively used in the domain of machine vision. Self-attention is a crucial element in transformer-based models. Furthermore, the vision transformer (ViT)^17^ differs from hierarchical transformers often used in computer vision. It functions as a resilient and non-hierarchical framework, serving as a foundational structure for image categorization. Traditionally, transformer models like Swin^18^, MViT^19^, PVT^20^, and PiT^21^ did not use ConvNet concepts like convolution and pooling. UViT^22^ used the breadth, depth, and input resolution of ViT models, together with a progressive attention mechanism, to proficiently manage high-resolution images. Carion et al.^23^ presented a framework named DETR for object identification. DETR employs a transformer-based approach. Wu et al.^24^ used ConvNet to extract visual tokens and get the representation. Later on, transformers were used to alter the extracted tokens and depict the relationships between them. Kobayashi et al.^25^ introduced channel attention blocks as a method for anomaly identification, with the purpose of emphasizing important channel information. The Partial Semantic Aggregation Vision Transformer, proposed in the article of Yao et al.^26^, is a scalable framework for anomaly identification in industrial movies. It allows simultaneous multi-category anomaly detection.

Bearing the above-mentioned analysis in mind, this study proposes an innovative transformer-based framework. The proposed framework is designed to effectively handle the intrinsic correlation in multi-views obtained from IoD in cropland scenarios. To reduce the global inductive bias, the proposed model is pre-trained using the large-scale ImageNet-ISLVRC dataset^27^ before fine-tuning it with manually-collected images from farmlands. An attention mechanism is also presented, including a dynamic attention module based on a shifting window. And a novel loss function is used to enhance the accuracy of anomaly detection. Through rigorous experiments using 6803 frames from farmland scenes, the proposed strategy demonstrates superiority over current deep-learning methods.

The contributions of this study includ the following:

- This is an early exploration of anomaly detection in cropland monitoring.
- A transformer-based model is proposed to realize the anomaly detection task.
- Rigorous assessments establish the excellence of this work compared to the most advanced algorithms and illustrate its resilience across different workloads.

The remaining of this paper is organized as follows: “Methods” section details the proposed methods, elaborating on the dataset, implementation steps, and key techniques involved. In “Results” section, the experimental results are presented, including the experimental settings, and a comprehensive analysis of the obtained outcomes. “Discussion” section is dedicated to the discussion, where the implications of the results are explored, and potential limitations are addressed. Finally, “Conclusion” section concludes the paper, summarizing the main findings, highlighting the contributions of the research, and suggesting directions for future work.

## Methods

### Dataset and image preprocessing

The Multi-View Vision Transformer (MVVT) introduced in this research was first trained using the ImageNet-ISLVRC database^27^, a publicly available dataset that has been widely used to enhance the precision of object detection and classification since 2010. The training dataset comprises 50,000 images, with each image assigned a label from a pool of 1000 categories. A total of 6803 frames were extracted from the images taken by 16 sets of drones (model: DJI JY03-4K; size: 31–40 cm; channels: 4; material: plastic). When capturing a image, the sRGB color space is chosen by lighting it with white fluorescent light. The collection is located inside the premises of a university campus in Zibo, Shandong Province, China. In general, the drones were arranged in a well-structured IoD system, where each drone was allocated a certain preplanned route to traverse across the campus. To maintain continuous drone operation, each drone was recharged every 15 min throughout the voyage and collected scene images every 30 s. The collected images have a resolution of 8192 pixels in width and 4096 pixels in height. After completing the data collection process, each sample frame was classified as either anomalous or normal using a majority vote approach carried out by three machine vision professionals. The dataset, consisting of 6803 image-label pairs, potentially contains missing pairs. To address this, a manual check was systematically performed to identify such missing pairs, and the reasons for their absence, such as data collection errors, were logged. For outlier detection, a combination of automated and manual methods was employed. Initially, automated screening was carried out to flag images with low resolution or abnormal dimensions. Subsequently, the research team manually inspected these flagged images to discriminate between genuine outliers and legitimate cases within the context of cropland scenarios. Regarding imbalanced data, simple random oversampling, which involved duplicating minority class samples, and undersampling, through randomly deleting majority class samples, were applied to balance the distribution of normal and abnormal cropland scenarios.

It should be mentioned that the images that were initially collected were labeled utilizing the annotation tool known as *LabelMe*. The images with added annotations were stored in the MS COCO format^28^. Both the images and the corresponding JSON file were generated. Furthermore, a sequence of modifications were implemented to the manually collected images in order to enhance their diversity. The changes included both horizontal and vertical mirroring, as well as rotation. It is important to observe that each image and its modified variants are classified under the same designation.

### The proposed multi-view vision transformer framework

Initially, a single-view vision transformer (ViT) was utilized. However, to better handle the local structures in cropland data, the shifted window structure from the Swin Transformer was incorporated, including W-MSA and SW-MSA. This change reduced computational complexity while enhancing local pattern recognition. Subsequently, considering the multi-view nature of the captured cropland data samples, a dual-view Swin Transformer was introduced to integrate complementary information from different perspectives, aiming to improve anomaly detection accuracy.

**Fig. 1:**
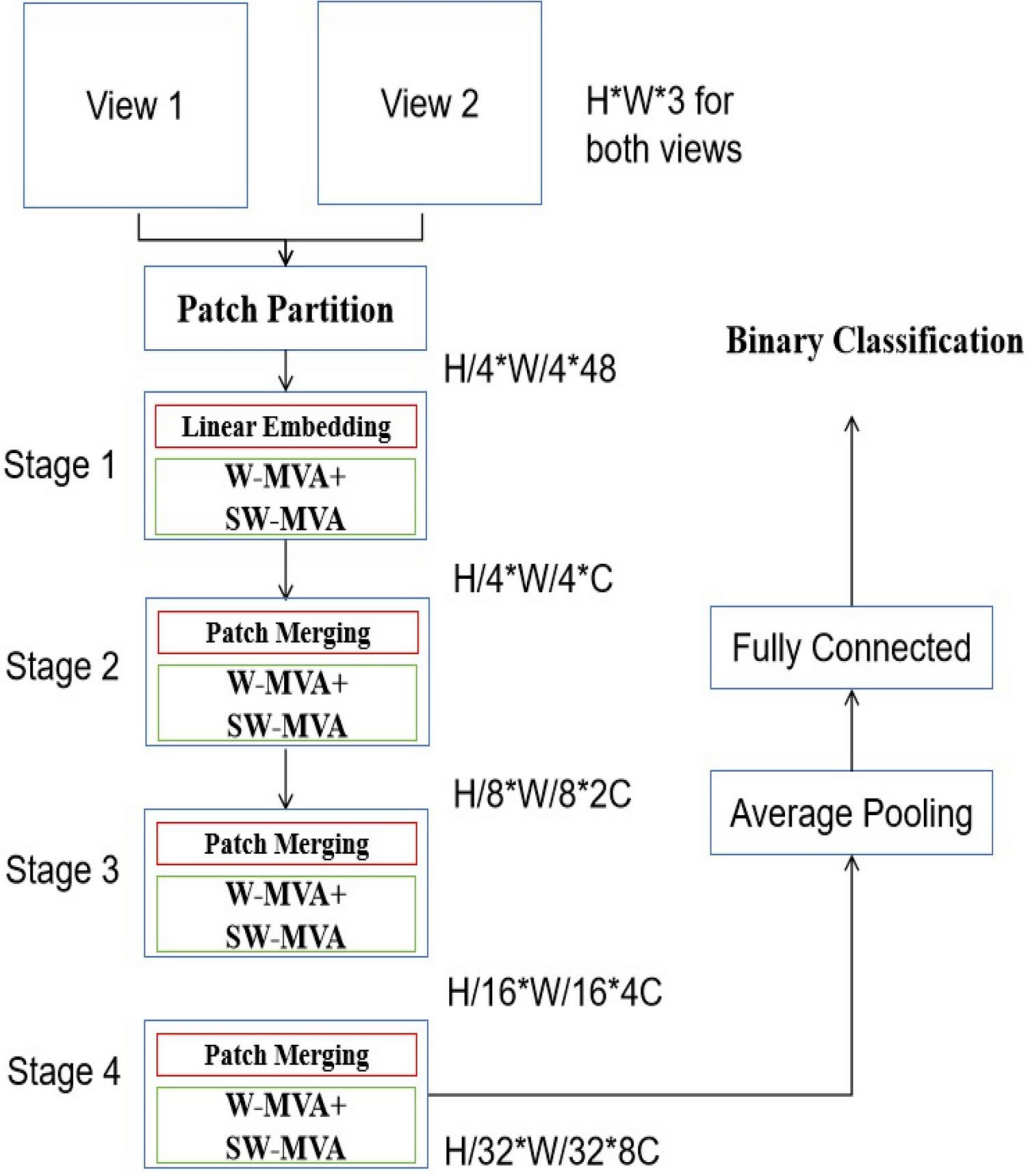
The proposed pipeline for anomaly detection in a cropland.

Then, the information about the suggested MVVT architecture for detecting anomalies in a farmland is provided. This model is constructed based on a vision transformer that incorporates shifting window and attention mechanics. It is worth mentioning that the attention mechanism has been used in several studies, such as Refs.^18–20^, either alone or in conjunction with convolutional layers. However, the analysis demonstrates that transformer-based models may provide comparable results to CNN and hybrid designs. The architecture of the proposed MVVT is shown in Fig. 1, and it is largely taken from the works of ViT^29^ and Swin^18^. The MVVT architecture takes the series of image patches as its input.

#### Input of the proposed model

The described model typically takes split image patches as input, which are obtained from images gathered by the drones. The suggested vision transformer incorporates position embeddings into its input to provide spatial information. In a real-life situation, each UAV begins its flight 30 s prior to acquiring 1 s of video footage. Throughout the journey, each UAV gathers video recordings at 30-s intervals. It is important to observe that the velocity of the UAVs is below 3 m per second, and the UAVs have the capability to capture images from distances beyond 100 m.

This model utilizes 2-dimensional (2D) embeddings for each view, following the vision transformer. In order to provide input to the suggested model, the input image is resized from $x\in {\mathbb {R}}^{H\times W\times C}$ into image patches represented as $x_p\in {\mathbb {R}}^{N\times (P^2\cdot C)}$. The variables *H*, *W*, and *C* indicate the height, width, and number of channels of an image, respectively. The variable *P* specifies the width and height of a patch. Next, the patches undergo a transformation and are converted into a vector with a length of *D*.

Like the vision transformer, a trainable class token is added to create a series of embeddings ($z_0^0=x_{class}$), and the resulting output from the transformer ($z_L^0$) is represented as *y*. Moreover, the position embedding is used to include the information about the location in addition to the sequence of patches.

1 $$\begin{aligned} z_0=[x_{class};x_p^1E; x_p^2E;...;x_p^NE]+E_{pos}, \end{aligned}$$

where $$E\in {\mathbb {R}}^{P^2\cdot C}\times D$$ and $$E_{pos}\in {\mathbb {R}}^{(N+1)\times D}$$.

#### Encoder

The previously stated $z_0$ serves as the input for the suggested transformer. Building upon the research conducted by Vaswani et al.^16^, the model being discussed considers the input patches as individual tokens. Each encoder has *L* layers, consisting of a multi-head self-attention (MSA) layer and a multi-layer perception (MLP) layer. In addition to the MSA module, a layerNorm module is used before to each block, and a residual block is employed after each block. A two-layer multilayer perceptron (MLP) with a Gaussian error linear unit (GELU) as the classification head is connected to the variable $z_L^0$.

The MSA module is based on the self-attention (SA) mechanism, as described in Ref.^16^. Semantic analysis is used to quantify the similarity between a query and its related keys, taking into account weighting values. Thus, the result may be derived by calculating the weighted total of all the values. More precisely, the input $Z\in R^{N\times D}$ consisting of N vectors of length D is leveraged.

2 $$\begin{aligned} {[}Q,K,V{]}=ZW_{QKV}, \end{aligned}$$

where $W_{QKV}$ denotes the weight matrix that can be updated by training. All of the weights are computed into the probabilities P with the following function:

3 $$\begin{aligned} P=softmax(\frac{QK^T}{\sqrt{D}}), \end{aligned}$$

where *D* is the length of each vector in Q, K, and V. Finally, the output of the SA mechanism can be mathematically expressed as:

4 $$\begin{aligned} SA(Z)=PV. \end{aligned}$$

Furthermore, the MSA mechanism employs the SA mechanism several times simultaneously, enhancing the extraction of information from the input for each head individually. The result of the MSA is the combination of all the components of the heads, which is represented as:

5 $$\begin{aligned} MSA(Z)={[}SA_1(Z);SA_2(Z);...;SA_h(Z){]}W_{MSA}, \end{aligned}$$

where *h* denotes the number of heads in the MSA module, and Z represents the feature map.

**Fig. 2:**
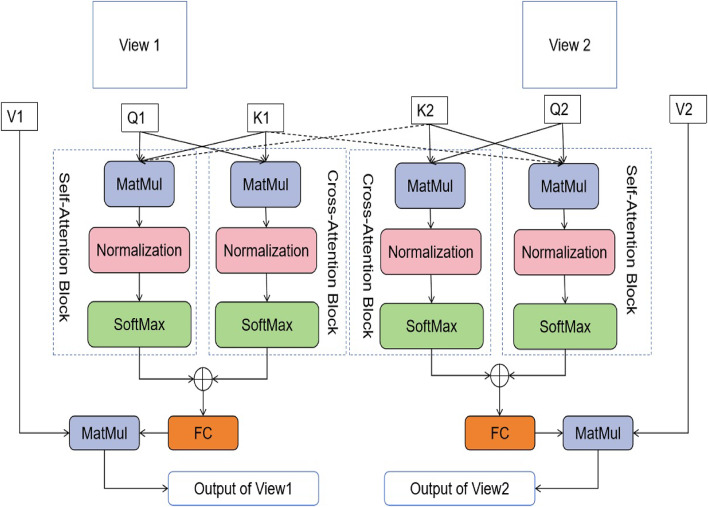
The self-attention and cross-attention modules provided in the proposed vision transformer.

In contrast to the MSA module, this research introduces the multi-view attention (MVA) module, seen in Fig. 2. Each view generates its associated Q, K, and V matrices. Furthermore, in order to record the connections between two perspectives, the K entries are moved between them. The output of the MVA is the combination of the output from both viewpoints, which is expressed as:

6 $$\begin{aligned} & MVA(Z)=<O_{view1},O_{view2}>, \end{aligned}$$

7 $$\begin{aligned} & O_{view1}=FC(\sigma (Q_{view1}.K_{view1}^T)\bigoplus \sigma (Q_{view1}.K_{view2}^T).V_{view1}, \end{aligned}$$

8 $$\begin{aligned} & O_{view2}=FC(\sigma (Q_{view2}.K_{view2}^T)\bigoplus \sigma (Q_{view2}.K_{view1}^T).V_{view2}, \end{aligned}$$

where *FC* denotes fully connected to provide the linear operation, $\sigma$ is the activation function, and $\bigoplus$ represents the concatenation operation. In addition, $Q_{view1}$, $K_{view1}$, $Q_{view2}$, $K_{view2}$, $V_{view1}$, and $V_{view1}$ represent the Q, K, V matrices for both views, respectively.

**Fig. 3:**
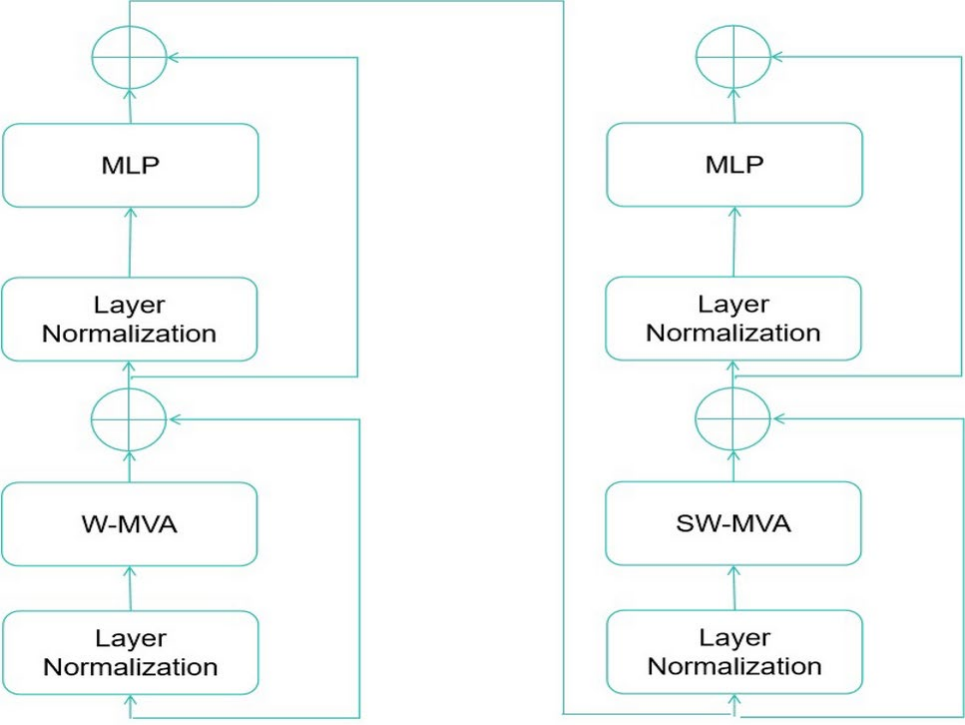
The successive W-MVA and SW-MVA modules.

Furthermore, taking inspiration from the research conducted by Swin^18^, both the regular windowing MSA (W-MSA) and shifted windowing MSA (SW-MSA) modules have been adapted into regular windowing multi-view attention (W-MVA) and shifted windowing multi-view dynamic attention (SW-MVA) modules, as seen in Fig. 2. It is important to mention that both the W-MVA and SW-MVA modules (as shown in Fig. 3) used the MVA module as the internal attention mechanism.

And the successive W-MVA and SW-MVA modules can be mathematically formulated as:

9 $$\begin{aligned} & Z_{l}^{\prime }=W-MVA(LN(Z_{l-1}))+Z_{l-1}, \end{aligned}$$

10 $$\begin{aligned} & Z_{l}=MLP(LN(Z_{l}^{\prime }))+Z_{l}^{\prime }, \end{aligned}$$

11 $$\begin{aligned} & Z_{l+1}^{\prime }=SW-MVA(LN(Z_{l}))+Z_{l}, \end{aligned}$$

12 $$\begin{aligned} & Z_{l+1}=MLP(LN(Z_{l+1}^{\prime }))+Z_{l+1}^{\prime }, \end{aligned}$$

**Fig. 4:**
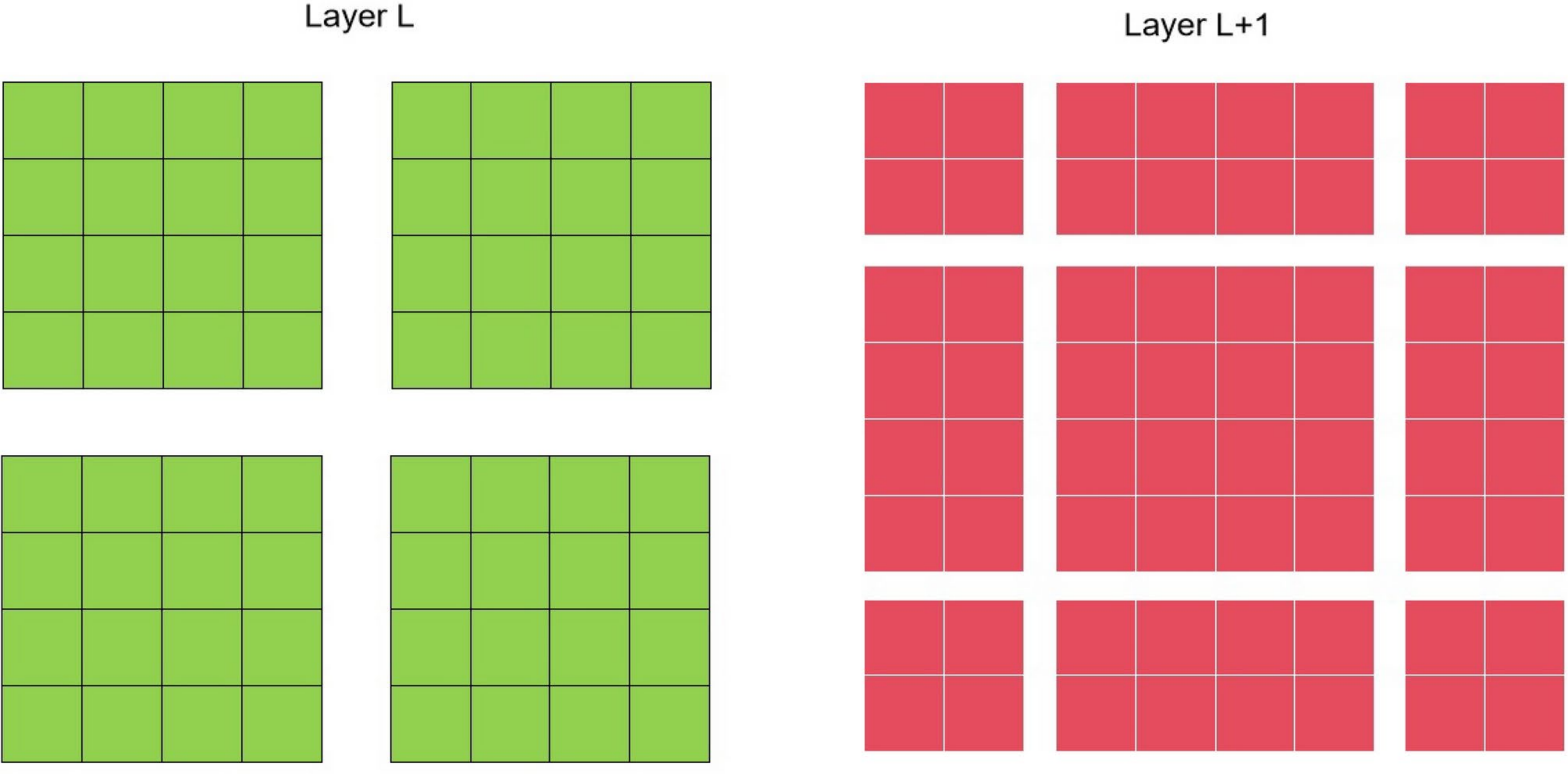
The diagram of the adopted shifted windowing mechanism. L denotes one specific layer in the proposed vision transformer.

To be specific, the shifted windowing mechanism adopted in the SW-MSA module is illustrated in Fig. 4.

Additionally, the vision transformer structure concludes with the use of a linear layer to combine the derived feature maps from both viewpoints.

13 $$\begin{aligned} y=Linear(LayerNorm{[}(Z_L^0)_{view1}+(Z_L^0)_{view2}{]}), \end{aligned}$$

where Linear(.) denotes a linear function, *L*=1 or 2, $(Z_L^0)_{view1}$ and $(Z_L^0)_{view2}$ represent the output of each view, respectively.

It is important to note that, unlike vision transformers, the vision transformer being discussed here receives the sequence of patches from distinct views individually. More precisely, the position embeddings represent the order of the image patches in two different views.

### Interpretability of the proposed approach

The proposed model, designed for cropland anomaly detection, commences with a comprehensive feature extraction process from multi-view drone-captured images. The layers in the proposed model are adept at discerning texture-based features. Accordingly, they can identify the fine-scale patterns in crop canopies, such as the presence of irregular leaf arrangements or abnormal growth directions, which might be indicative of crop stress or disease. At the classification stage, the model assigns a probability score to sub-region within the image. And this score is based on the aggregated information from the previously extracted features.

## Results

### Implementation details

In summary, the images from ImageNet-ISLVRC^27^ were used to perform the first training of the proposed transformer model. Moreover, the main configurations consist of using RMSprop as the optimizer, setting the learning rate to 0.002 with a reduction factor of 0.2, and using a batch size of 16 images. This was accomplished by using PyTorch^30^ and 4 NVIDIA Telsa V100 GPUs equipped with 64GB of HBM2 memory each.

Firstly, the influence of three factors, namely layers (L), model size (D), and number of heads (h), was investigated on the proposed transformer. This was accomplished by using a subset of the whole data sets. The pre-training of the transformer model was performed using the ImageNet-ISLVRC dataset^27^ by using the optimal parameter combination. In addition, the manually collected image samples were exploited to further enhance the recommended transformer via the process of fine-tuning. In addition, the comparison tests were conducted between the deep learning models and the proposed technique. The results indicate that the proposed transformer model surpasses the existing state-of-the-art models in terms of performance metrics like sensitivity, specificity, accuracy, and F1 score. Ultimately, the ablation study were carried out to evaluate the effectiveness of the suggested model. In addition, to implement overfitting mitigation, both 10-fold, 15-fold, and 20-fold cross-validation techniques were leveraged in the experiments.

### Loss function

The transformer-based pipeline utilizes the integration of multi-view components as the loss function.

14 $$\begin{aligned} Loss_{model}=Loss_{view1}+Loss_{view2}, \end{aligned}$$

where $Loss_{view1}$ and $Loss_{view2}$ denote the multi-view cross-entropy loss, respectively. By adding a penalty term to the loss function, L2 regularization helps in reducing the complexity of the model by shrinking the weights. This also prevents the model from over-emphasizing on specific patterns in the training data.

### Evaluation metrics

The parameters used for assessment in this research are sensitivity, specificity, accuracy, and F1 score. Specifically, sensitivity measures the model’s ability to identify actual abnormal cropland areas (positive cases). In cropland monitoring, high sensitivity can prevent the omission of key anomalies and reduce crop losses. Specificity is used to evaluate the model’s ability to correctly identify normal cropland areas (negative cases). High specificity can effectively reduce the misjudgment of normal cropland as abnormal and avoid waste of resources. Accuracy reflects the overall correctness of the model’s predictions. It can intuitively demonstrate the model’s comprehensive ability to judge the normal and abnormal states of cropland. F1-score can comprehensively evaluate the model’s performance in detecting anomalies. A high F1-score indicates that the model performs well in both identifying positive cases and controlling false alarms. The metrics used in the trials can be characterized as follows:

- Sensitivity: The ratio between true positives (TP) cases and $(TP + FN)$, where *FN* denotes false negative.
  15 $$\begin{aligned} Sensitivity=\frac{TP}{TP+FN}. \end{aligned}$$
- Specificity: The ratio between the true negatives (TN) and $(TN+FP)$, where *FP* denotes false positives.
  16 $$\begin{aligned} Specificity=\frac{TN}{TN+FP}. \end{aligned}$$
  There is a trade-off between sensitivity and specificity. Increasing sensitivity makes the model more likely to judge a sample as positive. Although it can capture more real-world anomalies, it may increase false alarms and reduce specificity. Conversely, increasing specificity makes the model more cautious in judging positive cases, which may miss some real-world anomalies and lead to a decrease in sensitivity.
- Accuracy:
  17 $$\begin{aligned} Accuracy=\frac{TP+TN}{TP+FN+TN+FP}. \end{aligned}$$
- F1 score:
  18 $$\begin{aligned} & F1 =2\times \frac{Precision \times Sensitivity}{Precision+Sensitivity}, \end{aligned}$$
  19 $$\begin{aligned} & Precision=\frac{TP}{TP+FP}. \end{aligned}$$

To note that in the cropland anomaly detection task, sensitivity is the most critical metric. Avoiding the omission of abnormal areas is crucial for ensuring crop yields and reducing economic and ecological losses. At the same time, it is also necessary to reasonably maintain specificity to control false alarms.

### Ablation study

**Fig. 5:**
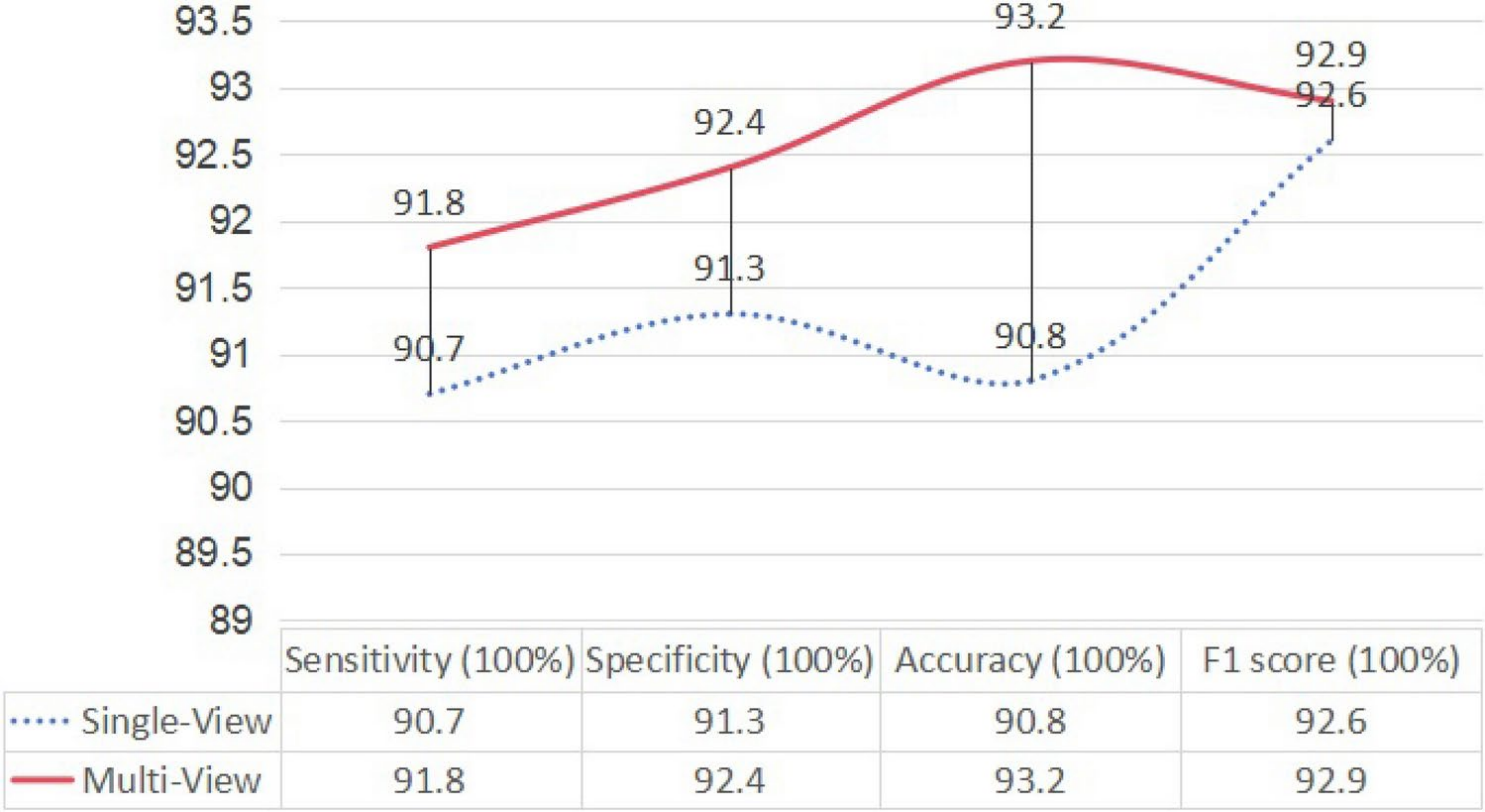
The comparison between the single view and multi-view architectures on 30% of the presented dataset.

Given that the suggested model is a hybrid design, the first step was to quantify the disparity between the individual view and the multi-view model. The discrimination performance was calculated using the single views, using 30% of the manually gathered dataset. As seen in Fig. 5, it is evident that the hybrid model outperforms the separate models in terms of sensitivity, specificity, accuracy, and F1 score.

In order to maximize the use of both intra-image and inter-image data, the concept of the multi-view transformer was proposed. Furthermore, in order to get a precise classification result, the newly developed loss function was presented. The benefit of the multi-view structure has been shown by the results of both the comparative trials and ablation studies.

In addition, Table 1 is provided to determine whether removing outliers from the dataset makes any difference for the proposed approach, using 30% of the manually gathered dataset.

**Table 1:** Comparison of the performance of the proposed approach before and after outlier removal.

Performance metric	Before outlier removal (%)	After outlier removal (%)
Sensitivity	83.9	91.8
Specificity	84.3	92.4
Accuracy	85.7	93.2
F1-score	84.1	92.9

### Influence of the hyper-parameters on the proposed transformer

The comparative tests were performed on a portion of the gathered images, testing different combinations of parameters to determine the most effective combination of the three parameters for the suggested model. Moreover, it is expected to provide an enhanced categorization outcome for the whole dataset.

As seen in table Table 2, the successive combinations of these three factors were evaluated. The indicated combinations are identified by the MVVT as initials and the hyper-parameters, which correspond to the actual parameter values.

**Table 2:** The combinations of the 3 parameters in the proposed transformer.

Combination	Layer (L)	Dimension (D)	Number of heads (h)
MVVT_2_64_4	2	64	4
MVVT_2_64_8	2	64	8
MVVT_2_128_4	2	128	4
MVVT_2_128_8	2	128	8
MVVT_4_64_4	4	64	4
MVVT_4_64_8	4	64	8
MVVT_4_128_4	4	128	4
MVVT_4_128_8	4	128	8

**Fig. 6:**
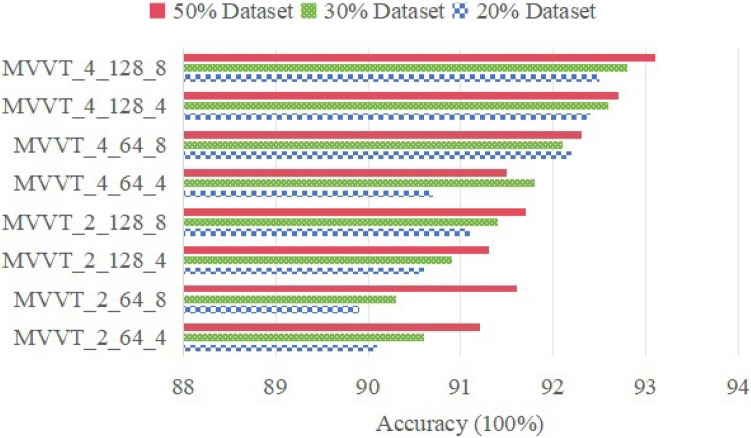
The influence of 3 parameters on the proposed transformer.

It is important to mention that, at this point, only three parameters were chosen. Monitoring more variations would be impractical. The transformer model obtained was a mix of MVVT_4_128_8, as seen by the comparison result in Fig. 6.

Furthermore, the comparative tests were performed including the mean square error (MSE) loss, cross entropy (CE) loss, and the suggested loss function (as seen in Table 3).

**Table 3:** Influence of various loss functions on the proposed approach.

Loss function	Sensitivity (%)	Specificity (%)	Accuracy (%)	F1 score (%)
MSE	90.7	89.8	90.2	89.7
CE	91.1	91.9	92.3	92.9
This work	92.8	93.1	93.5	94.1

### Comparison between the state-of-the-arts and the proposed transformer

The suggested technique demonstrates exceptional performance in terms of sensitivity, specificity, accuracy, and F1 score, as seen in Table 4. This implies that the proposed technique may have greater benefits compared to the present leading methods in the field of anomaly detection.

**Table 4:** Performance comparison between state-of-the-art techniques and this work in terms of sensitivity (%), specificity (%), accuracy (%), and F1 score (%).

Methods	Sensitivity	Specificity	Accuracy	F1 score
U-Net^31^	81.2	82.4	83.7	81.9
Mask R-CNN^32^	81.9	82.5	82.9	80.6
ExtremeNet^33^	81.7	82.3	83.6	82.3
TensorMask^34^	82.2	82.9	84.3	81.8
Visual transformer^24^	89.5	85.6	87.3	86.2
ViT^17^	88.5	86.4	87.1	87.1
MViT^19^	87.9	87.5	88.1	87.9
PiT^21^	87.6	88.2	89.4	88.3
PVT^20^	89.3	89.2	90.1	89.5
UViT^22^	88.6	89.7	91.5	90.3
Swin transformer^18^	89.1	87.2	88.0	87.4
Proposed work (10-fold)	92.8	93.1	93.5	94.1
Proposed work (15-fold)	91.7	92.5	92.9	93.4
Proposed work (20-fold)	90.4	91.6	92.3	92.8

Specifically, the single-view ViT had a sensitivity of 88.5%, specificity of 86.4%, accuracy of 87.1%, and an F1-score of 87.1%. After adopting the Swin Transformer, these metrics increased. The proposed approach (10-fold) achieved a sensitivity of 92.8%, specificity of 93.1%, accuracy of 93.5%, and an F1-score of 94.1%, demonstrating the effectiveness of the proposed method enhancements.

Furthermore, different starting weights of random initialization were evaluated during the training process of the proposed suggested transformer on ImageNet-ISLVRC. The deterministic strategy achieves convergence in less than 15 epochs, whereas the stochastic set requires more than 30 epochs. using the other hand, the transformer trained using Image-ISLVRC has a higher starting value, and the differences in losses follow a regular pattern.

Moreover, the confusion matrices of the anomaly detection task using the competing methods, are provided in Table 5. The confusion matrix serves as a critical instrument for visualizing the performance of the competing models.

**Table 5:** Confusion matrices for the competing methods.

Methods	TP	FP	FN	TN
U-Net	2762	599	640	2802
Mask R-CNN	2787	593	615	2808
ExtremeNet	2773	600	629	2801
TensorMask	2797	580	605	2821
Visual transformer	3045	490	357	2911
ViT	3013	462	389	2939
MViT	2991	425	411	2976
PiT	2980	402	422	2999
PVT	3038	367	364	3034
UViT	3018	349	384	3052
Swin transformer	3031	435	371	2966
Proposed (10-fold)	3158	234	244	3167
Proposed (15-fold)	3119	255	283	3146
Proposed (20-fold)	3076	285	326	3116

## Discussion

This research presents a novel multi-view anomaly detection approach specifically designed for low-altitude circumstances. This is another instance when a visual transformer-based algorithm is used in settings with low altitude. The experimental results suggest that the suggested transformer may provide accurate detection results by leveraging its hybrid design.

It is important to mention that the vision transformer acts as the basis for the suggested anomaly detection system. Unlike other anomaly identification approaches published in the literature, the proposed system has the ability to include information from both viewpoints in the input image samples. Although the provided technique utilizes an end-to-end learning approach, more improvements are necessary to properly meet the objectives of anomaly detection. By using the attention mechanism often used in transformer-based methods, it is feasible to identify the relationships between global pixels in a image captured by UAVs. The experimental results provide proof that the suggested approach may guarantee the effectiveness of anomaly detection. Consequently, it functions as a good instrument for monitoring orchard.

Furthermore, compared with traditional multi-view methods based on convolutional neural networks, the vision transformer architecture in this study abandons local convolutional operations and divides the input multi-view images into patches and processes them as sequences. By using the self-attention mechanism, it can directly model the relationships between patches of each view, effectively integrating information from different perspectives and constructing a more comprehensive feature representation. In contrast, CNN-based methods have limitations in capturing long-range dependencies across views. In terms of feature extraction, many similar methods rely on hand-crafted features, while this multi-view vision transformer adopts an end-to-end learning approach to automatically explore complex patterns and relationships in multi-view data. Taking cropland images as an example, traditional methods rely on predefined rules to extract features, while this model can learn subtle features such as the co-occurrence of crop growth patterns in different views through its self-attention layers. In addition, when processing multi-view data, some methods simply concatenate the features of each view at an early stage and then process them jointly. However, this study uses a cross-attention mechanism to explicitly model the interactions between different views, enabling the model to selectively focus on relevant information from each view according to the task requirements. Compared with the early-stage concatenation methods, it can more effectively utilize the complementary information between views and improve the performance of the model.

Moreover, the proposed approach encounters several limitations that warrant attention. Firstly, the computational requirements of the proposed model are substantial. Training on the 6803-frame farmland dataset consumed a significant amount of time on a high-end GPU. This not only hampers the practical implementation in real-time cropland monitoring, where quick results are crucial for timely intervention, but also poses challenges for large-scale deployments. In such scenarios, multiple drones may be collecting data simultaneously, and the high computational load could lead to bottlenecks in data processing and analysis. Secondly, the model’s performance in complex and rare anomaly scenarios is sub-optimal. The mis-classification shows that the proposed model fails to accurately identify the complex situations. This can be mainly attributed to the limited presence of such complex scenarios in the training data. With only a small proportion of the training data representing these intricate anomalies, the model lacks sufficient exposure to learn and generalize effectively. Accordingly, the advantages and disadvantages of the proposed method are provided in Table 6.

**Table 6:** Advantages and disadvantages of the proposed method.

Aspect	Details
Advantages	Can handle intrinsic correlation in multi-views effectively
	Unique attention mechanism for better feature focusing
	Novel loss function improves accuracy
Disadvantages	High computational requirements
	Sub-optimal performance in complex and rare anomaly scenarios due to limited training data

## Conclusion

This study aimed to address the crucial issue of anomaly detection in cropland monitoring within the low-altitude security domain, leveraging the IoD. This research introduced a novel approach that significantly deviates from existing methods.

In the future, the potential of the proposed framework will be explored. This includes integrating data from other types of sensors, such as thermal, infrared, or LIDAR, to enhance the robustness and accuracy of anomaly detection across diverse environmental conditions. Additionally, efforts will be made to make the vision transformer model more interpretable and explainable, fostering trust and transparency in security-critical applications. Next steps also aim to enhance the deep-learning pipeline to reduce computational complexity and latency, making it more suitable for real-time anomaly detection in resource-constrained environments.

## Data Availability

The ImageNet-ISLVRC database used in this study can be downloaded from https://www.kaggle.com/c/imagenet-object-localization-challenge/data. The data that support the findings of this study are available from the corresponding author upon reasonable request.
